# Unexplained Giant Esophageal Ulceration in a Pregnant Woman With Severe Immunosuppression

**DOI:** 10.7759/cureus.102311

**Published:** 2026-01-26

**Authors:** Abay A Gobezie, Mekdem Bisrat, Samrawit W Zinabu, Elizabeth Beyene, Mrinalini Deverapal, Sneha Adidam, Farshad Aduli, Miriam Michael

**Affiliations:** 1 Internal Medicine, Howard University Hospital, Washington DC, USA

**Keywords:** esophago-gastro-duodenoscopy, human immunodeficiency virus (hiv) infection, idiopathic esophageal ulcer, pregnancy, severe immunosuppression

## Abstract

Esophageal ulceration is a well-recognized cause of morbidity in patients with advanced human immunodeficiency virus (HIV) infection, most commonly from opportunistic infections such as cytomegalovirus, herpes simplex virus, or Candida species. Idiopathic esophageal ulceration (IEU) is a diagnosis of exclusion associated with profound immunosuppression, typically with CD4 counts <50 cells/mm³. Its occurrence during pregnancy, particularly in congenital HIV infection, is exceedingly rare and presents unique diagnostic and therapeutic challenges.

We report a 32-year-old pregnant woman at 21 weeks' gestation with congenital HIV/AIDS and longstanding nonadherence to antiretroviral therapy (ART) presenting with progressive odynophagia and dysphagia. Evaluation revealed severe immunosuppression (CD4 count 4 cells/mm³, HIV viral load 96,000 copies/mL). Esophagogastroduodenoscopy showed multiple large, deep esophageal ulcers. Histopathologic evaluation with special stains and immunohistochemical studies excluded viral, fungal, and mycobacterial etiologies. HIV-associated IEU was diagnosed. Re-initiation of ART with supportive care resulted in symptomatic improvement within four weeks and subsequent cesarean delivery following preterm labor.

This case highlights the importance of early endoscopic evaluation in severely immunocompromised pregnant patients with persistent esophageal symptoms and underscores the critical role of immune reconstitution through ART in managing IEU.

## Introduction

Esophageal disease is an important cause of morbidity and mortality in patients with human immunodeficiency virus (HIV) infection [1]. Up to 40% of patients with acquired immunodeficiency syndrome may develop symptoms of esophageal disease [2]. The etiology of HIV-related esophageal ulceration varies. After all known etiologies are excluded, a subgroup of patients remains with esophageal ulceration known as idiopathic esophageal ulceration (IEU) [1]. Esophageal ulcers in immunocompromised patients, especially those with advanced HIV/AIDS, with a prevalence of <10%, are commonly due to opportunistic infections such as cytomegalovirus (CMV), herpes simplex virus (HSV), or Candida.

Idiopathic HIV-associated giant esophageal ulcers correlate with profound immunosuppression (CD4 <50 cells/mm³) and cause significant morbidity. IEU is strictly a diagnosis of exclusion, made only after a comprehensive evaluation rules out infectious, medication-induced, and other identifiable causes. Although traditionally considered an uncommon diagnosis, HIV-associated IEU may occur more frequently than previously recognized. In a small cohort study of 51 patients with HIV, nearly half (49%) of identified esophageal ulcers were idiopathic, compared with an estimated prevalence of approximately 1.1% among immunocompetent individuals [3].

The occurrence of IEU during pregnancy, particularly in patients with perinatally acquired HIV, is exceedingly rare and poorly documented in the literature. Pregnancy introduces additional diagnostic and therapeutic complexity, as physiological changes can mask or mimic esophageal symptoms, and concerns about fetal safety limit diagnostic and treatment options. A significant knowledge gap exists regarding the optimal diagnostic approach and management of IEU in pregnant women with severe immunosuppression. The scarcity of reported cases of IEU in pregnant patients with congenital HIV makes each case valuable for expanding clinical understanding. Here, we present a rare case of HIV-associated giant IEU in a pregnant woman with perinatally acquired HIV, highlighting the diagnostic complexity and the importance of early endoscopic evaluation. The abstract of this case report was previously presented at the American College of Gastroenterology (ACG) on October 28, 2024.

## Case presentation

A 32-year-old pregnant woman (G3P1102 at 21 weeks' gestation) presented with a three-week history of progressive odynophagia and dysphagia to both solids and liquids. She denied hematemesis, melena, fever, or weight loss. Her medical history included congenital HIV/AIDS with a history of prolonged nonadherence to antiretroviral therapy (ART), and she was on nifedipine.

**Figure 1:**
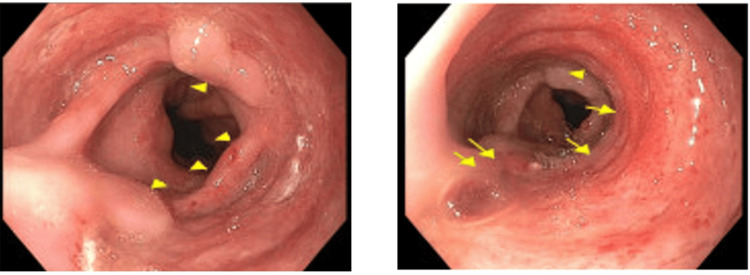
Upper endoscopy findings of multiple giant idiopathic esophageal ulcers. Arrows indicate giant esophageal ulcers.

On presentation, vital signs at triage were notable for a blood pressure of 126/67 mmHg, pulse rate of 107 beats per minute, respiratory rate of 18 breaths per minute, temperature of 98.6°F, and oxygen saturation of 100% on room air. She was in no acute distress. On physical examination, she was 39 kg with significant cachexia and bitemporal wasting. Abdominal examination revealed a gravid, non-tender uterus. Fetal heart rate was reassuring. Obstetric anatomy ultrasonography demonstrated findings suggestive of fetal growth restriction (estimated fetal weight <6th percentile). Laboratory evaluation revealed profound immunosuppression with a CD4 count of 4 cells/mm³ and an HIV viral load of 96,000 copies/mL. Given her persistent symptoms and severely immunocompromised state, esophagogastroduodenoscopy (EGD) was performed. Endoscopic evaluation revealed multiple large, deep, cratered esophageal ulcers without active bleeding or stigmata of recent hemorrhage (Figure 1).

**Figure 2:**
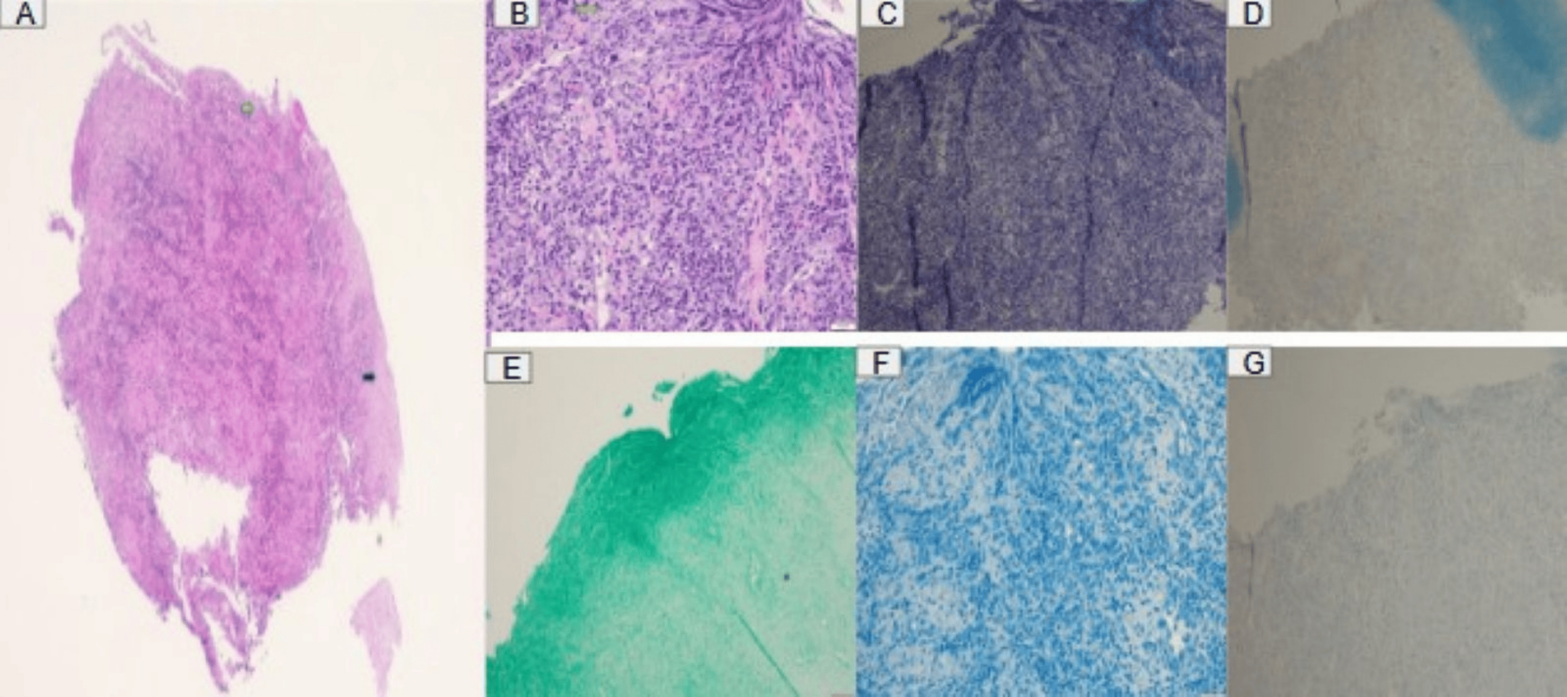
Histopathology (A-B) and immunohistochemical staining (C-G) excluding opportunistic infectious etiologies. A. Squamous epithelium and loss of squamous surface at the site of ulceration. B. Infiltration of acute inflammatory cells, histiocytes, and reactive epithelial cells that can mimic viral inclusions. C. Negative CMV immunostaining. D. Negative HSV immunostaining. E. Negative GMS special stain for fungal infections. F. Negative AFB special stain for mycobacterial organisms. G. Negative adenovirus immunostaining. AFB: acid-fast bacilli, CMV: cytomegalovirus, GMS: Gomori methenamine silver, HSV: herpes simplex virus.

Histopathologic examination demonstrated squamous mucosa with diffuse ulceration, acute and chronic inflammatory infiltrates, and granulation tissue formation (Figure 2). Special stains and immunohistochemical studies were negative for CMV, HSV, Candida species, and acid-fast bacilli, effectively excluding opportunistic infectious etiologies.

Based on the clinical presentation, endoscopic findings, severe immunosuppression, and exclusion of infectious causes, a diagnosis of HIV-associated IEU was made. The patient was re-initiated on ART with close infectious disease and obstetric follow-up. Her dysphagia improved after four weeks, and she subsequently delivered via cesarean section following preterm labor.

## Discussion

IEU occurs in 10-40% of HIV patients with esophageal symptoms, predominantly when CD4 counts fall below 50 cells/mm³ [4,5]. The pathophysiology involves direct HIV viral cytopathic effects, immune-mediated mucosal injury, and dysregulated cytokine production, though precise mechanisms remain incompletely understood [6].

HIV envelope glycoprotein gp120 induces direct epithelial cytotoxicity through CD4-independent mechanisms [7]. Elevated levels of TNF-α (tumor necrosis factor-α), IL (interleukin)-1, and IL-6 impair epithelial barrier function and delay mucosal healing [8]. Diagnosis requires exclusion of opportunistic pathogens through comprehensive microbiological and histopathological examination.

Differential diagnosis includes CMV esophagitis (large shallow ulcerations with viral inclusions), HSV esophagitis (multiple small vesicles with multinucleated giant cells), Candida esophagitis (white plaques with fungal hyphae on periodic acid-Schiff (PAS)/Gomori methenamine silver (GMS) staining), mycobacterial infection, drug-induced esophagitis, gastroesophageal reflux disease (GERD), and malignancy [9,10]. CMV and HSV are identified through immunohistochemistry or in situ hybridization. Histopathology reveals nonspecific squamous epithelial ulceration with granulation tissue, mixed inflammatory infiltrates, and reactive epithelial changes [11]. Absence of viral cytopathic effects, fungal elements, acid-fast bacilli, and malignant cells on routine and special staining is diagnostic. Immunohistochemical studies for CMV pp65, HSV-1/2, and viral nucleic acid in situ hybridization exclude infectious etiologies. CD8+ T lymphocyte predominance may be present but lacks specificity.

IEU occurrence during pregnancy in congenital HIV infection presents unique challenges. Pregnancy-associated Th2 cytokine shifts and reduced cell-mediated immunity may alter disease manifestations [12]. EGD can be safely performed during the second trimester when clinically indicated [13]. Management requires careful risk-benefit assessment, balancing maternal safety, fetal well-being, and teratogenic medication concerns.

Management centers on immune reconstitution through optimized ART, typically comprising two nucleoside reverse transcriptase inhibitors (NRTIs) with an integrase strand transfer inhibitor (INSTI) or protease inhibitor (PI). Symptom resolution occurs within two to four weeks, with endoscopic healing requiring four to eight weeks [4]. Adjunctive systemic corticosteroids (prednisone 40 mg daily, tapered over two to four weeks) accelerate symptom resolution by modulating inflammation [6]. Thalidomide (200-400 mg daily) demonstrates efficacy in refractory cases through TNF-α inhibition but is contraindicated in pregnancy [14]. Supportive care includes nutritional optimization, viscous lidocaine, proton pump inhibitors, and consideration of total parenteral nutrition in severe cases.

Prognosis is favorable with appropriate ART, though complications warrant monitoring. Hemorrhage may require endoscopic hemostatic interventions. Esophageal stricture formation may necessitate endoscopic dilation. Perforation, though rare, demands emergent surgical consultation. Long-term outcomes correlate with virological control and sustained ART adherence.

## Conclusions

This case describes a pregnant woman with congenital HIV infection and poor ART adherence who presented with progressive odynophagia and dysphagia. Endoscopic evaluation revealed multiple large esophageal ulcers, and an extensive infectious workup was negative, leading to a diagnosis of idiopathic HIV-associated esophageal ulceration. Clinical improvement occurred after re-initiation of ART, followed by delivery after preterm labor.

This case highlights the importance of considering IEU in immunocompromised patients when infectious causes are excluded. Early endoscopic evaluation and multidisciplinary management are essential, particularly in pregnant patients, as prompt immune reconstitution through ART can lead to symptom resolution and favorable maternal outcomes.
